# Hypothermia for Cardioprotection in Acute Coronary Syndrome Patients: From Bench to Bedside

**DOI:** 10.3390/jcm13185390

**Published:** 2024-09-12

**Authors:** Nikolaos Pyrpyris, Kyriakos Dimitriadis, Panagiotis Iliakis, Panagiotis Theofilis, Eirini Beneki, Dimitrios Terentes-Printzios, Athanasios Sakalidis, Alexios Antonopoulos, Konstantinos Aznaouridis, Konstantinos Tsioufis

**Affiliations:** First Department of Cardiology, School of Medicine, National and Kapodistrian University of Athens, Hippokration General Hospital, 115 27 Athens, Greece; npyrpyris@gmail.com (N.P.); panayiotisiliakis@gmail.com (P.I.); panos.theofilis@hotmail.com (P.T.); e.beneki@hotmail.com (E.B.); dimitristerentes@yahoo.gr (D.T.-P.); asakalidis@gmail.com (A.S.); antonopoulosal@yahoo.gr (A.A.); conazna@yahoo.com (K.A.); ktsioufis@gmail.com (K.T.)

**Keywords:** myocardial infarction, reperfusion injury, hypothermia, intracoronary hypothermia, ST-elevation myocardial infarction, acute coronary syndrome

## Abstract

Early revascularization for patients with acute myocardial infarction (AMI) is of outmost importance in limiting infarct size and associated complications, as well as for improving long-term survival and outcomes. However, reperfusion itself may further damage the myocardium and increase the infarct size, a condition commonly recognized as myocardial reperfusion injury. Several strategies have been developed for limiting the associated with reperfusion myocardial damage, including hypothermia. Hypothermia has been shown to limit the degree of infarct size increase, when started before reperfusion, in several animal models. Systemic hypothermia, however, failed to show any benefit, due to adverse events and potentially insufficient myocardial cooling. Recently, the novel technique of intracoronary selective hypothermia is being tested, with preclinical and clinical results being of particular interest. Therefore, in this review, we will describe the pathophysiology of myocardial reperfusion injury and the cardioprotective mechanics of hypothermia, report the animal and clinical evidence in both systemic and selective hypothermia and discuss the potential future directions and clinical perspectives in the context of cardioprotection for myocardial reperfusion injury.

## 1. Introduction

Coronary artery disease (CAD) is the most frequent cause of death and disability worldwide, responsible for over 7 million deaths per annum [1]. Recently, the Global Disease Burden data analysis showed that, globally, ischemic heart disease is responsible for 98.4 deaths per 100,000 persons, with similar rates in Europe and the United States [2]. Acute coronary syndrome (ACS) is the most important manifestation of CAD and is singlehandedly implicated in a major percentage of CAD-related mortality. The latest analyses show that ACS-related mortality is higher in men compared to women, including patients under 70 years of age, with these events progressively being reduced throughout the years by more than 50%, given the emergence of effective, rapid invasive treatment, as well as post-ACS pharmacotherapy [3]. Regarding the types of ACS, it is well known that they are categorized as ST-segment elevation myocardial infarction (STEMI), non-STEMI (NSTEMI), and unstable angina. More recent evidence shows that there is an increase in the proportion of NSTEMI, compared to STEMI, in both the United States [4] and Europe [5]. Greek registry data also report a 56.8% rate for NSTEMI and 43.2% rate for STEMI [6], with similar trends being showed by Spanish registries as well [7].

Primary percutaneous coronary intervention (pPCI) is the treatment of choice for patients presenting with STEMI, as suggested by international guidelines [8,9]. The aim of “as-soon-as-possible” revascularization of a STEMI patient is to restore blood flow and to limit the infarct size (IS), especially considering myocardial areas that are still viable in the segment of the occluded coronary artery. It has been shown that a reduction of the IS leads to lower adverse events post-revascularization, including mortality and heart failure (HF) hospitalizations [10,11]. However, as in other organs, reperfusion of the occluded segment may result in further damage of the surviving myocytes, an entity known as myocardial reperfusion injury. This pathology was first described in 1960 in an animal histology study, examining a series of reperfused myocardia, by Jennings et al., which noted cellular swelling, myofibril contracture, sarcolemma disruption, and intramitochondrial calcium phosphate molecules following coronary artery reperfusion [12]. Since this discovery, and as studies showcase that myocardial reperfusion injury may contribute up 50% of the IS, several investigators have aimed to identify pathogenetic relations as well as methods to limit this effect of revascularization, including hypothermia. Thus, we aim to provide a narrative review on this topic, searching the medical literature databases (Medline, ClinicalTrials.gov) and including the terms “systemic hypothermia or cooling, intracoronary hypothermia or cooling, myocardial reperfusion injury, myocardial infarction, acute coronary syndrome”. This review is going to delve into the pathophysiology of myocardial reperfusion injury and the data behind the potential use of hypothermia in the setting of STEMI, as well as discuss the combined use of hypothermia with other common cardioprotective options and clinical perspectives.

## 2. Pathophysiology of Myocardial Reperfusion Injury

Since the first report of myocardial reperfusion injury in the 1960s, a lot of work has been done to investigate the pathophysiology of this phenomenon, which may limit the benefit of revascularization. Myocardial reperfusion injury is a complex entity, with several molecular pathways contributing towards myocardial damage.

### 2.1. Key Pathophysiological Concepts

Several pathways during the early post-revascularization period, physiologically aiming to protect myocardial cells from the dreadful consequence of oxygen deprivation, are exaggerated and result in myocardial damage. Following reperfusion and reoxygenation of the occluded artery segment, an increase in reactive oxygen species (ROS) is being observed, in greater values than those generated by ischemia alone, leading to local toxicity [13,14]. Following ischemia, cardiomyocytes switch to anaerobic glycolysis and the accumulation of lactate, which decreases pH and creates an ion imbalance, which homeostatic mechanisms aim to restore. Specifically, an initial increase in natrium results from the activation of the Na^+^-H^+^ ion exchanger and deactivation of the Na^+^-K^+^ ATPase due to absence of energy (ATP); the Na^+^-Ca^2+^ exchanger tries to correct this, thus resulting in an efflux of Ca^2+^ in the myocardial cells [15]. The influx of Ca^2+^ is further increased in the immediate period post-revascularization, as the normalization of extracellular pH leads to corrective responses, especially regarding Na^+^ and Ca^2+^, thus compromising myocardium survival [16].

On a cellular level, these ion imbalances promote cellular swelling, loss of structural integrity and myofibril hypercontracture^+^ [17]. The presence of ATP and Ca^2+^ in the reperfused myocardium leads to activation of the myocardial fibrils and excessive contracture, which can result to myocardial death, as shown in histology studies (contraction band necrosis pattern) [18,19]. Mechanistically, Ca^2+^ oscillations have been found to have a causal relationship with hypercontracture, as their inhibition limits these effects [20,21]. Moreover, along with sarcoplasmic Ca^2+^ accumulation, changes in the mitochondrial membrane also result to mitochondrial Ca^2+^ efflux, with damaging effects in the mitochondrial function [22]. In particular, Ca^2+^ influxes towards the mitochondria via the mitochondrial Ca^2+^ transporters. This results in the loss of function of the mitochondrial permeability transition pore (MPTP), leading to mitochondrial membrane depolarization, uncoupling of the oxidative phosphorylation, depletion of myocardial ATP and myocardial death [23,24,25]. Furthermore, structural integrity, and, thus, resistance to mechanical stress, is compromised via Ca^2+^-activated proteases (calpains) that are activated post-pH normalization and, therefore, in the post-revascularization time frame [26]. Finally, these pathways are regulated and related to several well-known molecular paths, such as the small ubiquitin-like modifier (SUMO), NF-κB, NLPR3 and micro-RNA molecules, which can be inhibited by hypothermia [27].

Inflammation is also recognized as a myocardial cell-independent mechanism of myocardial reperfusion injury [28]. Both myocardial resident and systemic leukocytes respond to post-ischemia myocardial cell death. When a sustained pro-inflammatory environment exists, this can result in further damage of the myocardial cells in the early hours after the event, impairing systolic function and promoting fibrosis [29]. Interestingly, genetic variables may also have a role in the extent of injury. It has been shown recently by Veltman et al. [30] that the C-type lectin domain family 4 member E (CLEC4E), which is a significant protein in sterile inflammation, could be of significance in regulating ischemic injury. Several other genes are also related, with major therapeutic implications [31,32,33].

### 2.2. Myocardial Reperfusion Injury Types

There are four types of myocardial reperfusion injury. The first type is myocardial stunning. Myocardial stunning was first described by Braunwald and Kloner in 1982 as the presence of prolonged, post-ischemic ventricular dysfunction following brief periods of non-lethal ischemia [34]. Approximately 50% of patients with STEMI will have myocardial stunning, with 22% having complete recovery and 36% partial recovery of the myocardial function after 3 months [35]. This pathology was first described in canine models, showing reversible myocardial injury, but not substantial evidence of necrosis [36,37], as well as evidence of ATP levels (biochemical) stunning [38]. In studies with PCI, balloon occlusion of a coronary artery also results in myocardial stunning] [39]. Importantly, Wdowiak-Okrojek et al. [40] recently showed that the largest improvements in the systolic longitudinal strain and its rate are observed in the first 2 days, with further, but less noticeable, improvements in the following 6 months. However, the recovery of the diastolic performance takes longer, with most of it being recovered after 7 days.

The second type of myocardial reperfusion injury is reperfusion arrhythmias. These arrhythmias mostly relate to ventricular arrhythmias and can be present in the form of premature ventricular complexes to ventricular fibrillation. The most common type, however, is accelerated idioventricular rhythm, with signs of origin from the post-ischemic, reperfused area [41]. Pathophysiologically, the role of calcium and ion imbalance is fundamental, with delayed afterdepolarization being the most plausible electrophysiological explanation [42,43]. Although accelerated idioventricular rhythm was earlier thought to be a positive sign of reperfusion, indicating the time that revascularization was achieved, more recent evidence has reported that ventricular arrhythmia bursts are independently associated with IS extent in optimally reperfused STEMI patients [44,45,46], even in the presence of optimal epicardial and microvascular reperfusion [47].

The third type of myocardial reperfusion injury is the no-flow phenomenon, or microvascular obstruction (MVO) [48]. No-flow is defined angiographically as reduced antegrade blood flow and impaired dye penetration into the myocardium. However, non-reflow has been better categorized with the use of cardiac magnetic resonance imaging as MVO, which better described the presence of microvascular dysfunction. Despite the terms non-reflow and MVO having been used interchangeably in the literature, studies show that the diagnosis of MVO is higher with CMR (67%) compared to angiography (36%), while CMR also allows better patient stratification [49]. MVO has been identified as a prognosticator of cardiovascular outcomes in STEMI patients [50]. Another manifestation of the no-reflow phenomenon includes intramyocardial hemorrhage (IMH), which represents capillary destruction and, therefore, in comparison to MVO, is irreversible [51]. Pathophysiologically, post-reperfusion microvascular dysfunction is related to a handful of mechanisms, including endothelial dysfunction, excessive ROS presence, distal embolization, tissue edema and swelling, inflammation, destabilization of interendothelial cell junctions and vascular permeability [52]. The effect of myocardial reperfusion injury in microvasculature has been largely evident from animal studies, studying both MVO and IMH [53,54].

The final type of myocardial reperfusion injury is lethal myocardial damage. This entity describes the inability of myocardial cells that have survived to manage the aforementioned pathophysiologic phenomena, thus resulting in cellular death [55]. For several years investigators debated the presence of such an entity, as there is no particular method to accurately evaluate the progress of myocardial necrosis and IS from the time of ischemia onset to the time of reperfusion. However, preprocedural and intraprocedural interventions, either invasive or pharmaceutical, that aimed towards IS reduction and reperfusion injury mediation, including hypothermia which is going to be analyzed below, were ultimately indicative of this non-reversible pathological process.

## 3. The Role of Systemic Hypothermia

There have been numerous modalities used in order to halt myocardial reperfusion injury, with several intervention not succeeding its role of IS reduction in humans, despite positive effects in preclinical studies, resulting in further research.

Several preclinical studies have validated the cardioprotective role of systemic hypothermia, reporting IS reduction in animal models of ischemia-reperfusion injury. Early investigations have reported that IS was correlated with changes in temperature, with lower temperatures being related to reduced IS [56]. Further studies of endovascular cooling in small animals showcased that hypothermia decreases IS, while it can delay osmotic fragility and hypercontracture [57]. Dae et al. [58], in a larger animal (pig) model, using endovascular heat exchange catheters used 20 min after coronary occlusion until 15 min after reperfusion, reported that cooling reduced IS and myocardium area at risk, compared to normal temperatures, while maintaining microvascular flow and myocardial cell viability. Several preclinical studies followed, preparing the grounds for human studies [59]. Interestingly, as will be described below, some investigators reported that hypothermia during ischemia, but not during reperfusion, exhibits cardioprotective effects, which holds several key implications regarding the proper timing of hypothermia initiation, considering that hypothermia cannot be applied at the time of myocardial infarction (MI) in human patients, as is the case in animal studies, therefore potentially losing some of the strong benefits observed when initiated during ischemia [60]. Regarding pathophysiology mechanisms, several pathways that cooling interferes with have been proposed. These include the reduction in hypercontracture [57], reduced rate of ATP utilization [61], attenuation of MVO [62,63], decreased cardiac work [64], protection from hypoxia and hypoxia-induced apoptosis [65], prevention of intracellular Ca^2+^ overload via inhibition of the Na^+^/Ca^2+^ exchanger [66], attenuation of ROS production and maintenance of mitochondrial homeostasis [67,68]. In order to establish, therefore, benefit in humans, a handful of clinical studies have been performed (Table 1).

COOL-MI I pilot was the first-in-human trial to evaluate the safety and efficacy of endovascular cooling in patients with MI undergoing pPCI [69]. Towards this purpose, Dixon et al. enrolled and randomized 42 consecutive patients with MI to primary PCI with or without endovascular cooling (with a target temperature of 33 °C). Cooling was successfully applied to 95% of the hypothermia arm patients (20 patients). The primary endpoint of the trial was MACEs at 30 days and secondary endpoints was the size of the infarct, assessed by SPECT at 30 days (% of the left ventricle); the investigators demonstrated that endovascular cooling performance was safe (MACEs occurred in 0% vs. 10%, *p* = NS), without any statistically significant effect on the infarct size (IS) (2% vs. 8% of the left ventricle, *p* = 0.80). Ly et al. [70] evaluated, in a NICAMI study, the safety and feasibility of another hypothermia system, Arctic Sun Energy Transfer Pads (Medivance), in patients undergoing primary PCI. Hypothermia was achieved through pads (surface modality), placed on the patient’s back, abdomen and thighs once they were at the emergency department with a target temperature of 34.5 °C. The investigators enrolled nine patients, in which surface cooling was successfully performed, with a mean total cooling duration of 267 min (pre- and post-procedure), and all patients underwent successful primary stenting, with an achieved TIMI grade 3 flow in all patients. Regarding safety, there were no device-related serious adverse events; moreover, infarct size was evaluated in all patients by SPECT and reported to comprise 23% of the left ventricle.

COOL-MI I pilot failed to show hypothermia’s efficacy over IS. In RAPID-MI-ICE [71], the investigators tried to assess the safety and efficacy of hypothermia by both cold saline and endovascular cooling catheter, in patients with ACS, undergoing pPCI. For this purpose, 20 patients with ACS were enrolled and randomized to hypothermia arm and control arm. It is quite important to note that body temperature under 35 °C was achieved in all patients, and more importantly, cooling’s application was not associated with any significant delay in door-to-balloon time (43 ± 7 min in the hypothermia arm vs. 40 ± 6 min in the control arm, *p* = 0.12). All patients underwent successful primary stenting with TIMI grade 3 flow achieved. It was the first RCT that reported efficacy of hypothermia over placebo regarding infarct size (evaluated with cardiac magnetic resonance (CMR) 4 days after the PCI)—infarct size normalized to myocardium at risk decreased by 38% in the hypothermia arm compared with the control group (*p* = 0.041)—accompanied by a significant reduction in troponin levels (*p* = 0.01).

CHILL-MI was a multi-center, prospective, RCT that enrolled 120 patients with ACS (and specifically STEMI), planned for pPCI and randomized them to hypothermia by cold saline and endovascular cooling or standard of care; the primary endpoint of the trial was IS as a percent of myocardium at risk (MaR), evaluated by CMR 4 days post procedure [72]. The achieved temperature in the hypothermia arm was 34.7 °C before reperfusion, without any significant door-to-balloon time delay between hypothermia and control arm. Regarding hypothermia efficacy, CHILL-MI did not achieve a significant reduction in the primary endpoint (median IS/MaR 40.5% in the hypothermia arm, 46.6% in the control arm, with a relative reduction of 13%, *p* = 0.15). However, the investigators demonstrated that the incidences of HF and mortality (possibly driven by the heart failure endpoint) at median 45 ± 15 days of follow-up were significantly lower in the hypothermia arm (3% vs. 14%, *p* < 0.05), with this being one of the first trials to imply a positive trend on prognosis. What the researchers suggested is that anterior STEMI could be the type of ACS that would benefit more from hypothermia therapy; however, future research, more specified in this population, was needed.

The Evaluation of Ultrafast Hypothermia Before Reperfusion in STEMI Patients (VELOCITY) trial was a prospective, multi-center RCT that evaluated the safety and efficacy of another hypothermia system, which achieves cooling via lavaging the peritoneal cavity with temperature-controlled lactated Ringer’s solution (peritoneal hypothermia) [73]. Therefore, Nichol et al. enrolled 54 patients with an ACS, randomizing them to a peritoneal hypothermia arm (n = 28) and a control arm (n = 26) before they underwent pPCI. The primary efficacy endpoint was IS, evaluated by CMR 3–5 days post-procedure, and the primary safety endpoint was the composite endpoint of death reinfarction, ischemia-driven target vessel revascularization, major bleeding, sepsis, pneumonia, peritonitis, severe arrhythmia or renal failure, 30 days post-procedure. It was the first study in which peritoneal cooling was evaluated in a randomized setting. Firstly, the target temperature of <34.9 °C was achieved in 89% of the enrolled patients; however, the median door-to-balloon times was modestly higher by 15 min in the hypothermia arm (*p* = 0.007). Secondly, regarding efficacy, hypothermia was not associated with decreased infarct size. Indeed, IS was non-significantly higher in the hypothermia arm compared to the control arm (17.2% ± 2.3 vs. 16.0% ± 6.1, *p* = 0.54). Finally, there were some serious safety concerns, as hypothermia was associated with a significantly increased composite safety endpoint [0 (0%) patients in the control group vs. 6 (21.4%) patients in the hypothermia group, *p* = 0.01]. To summarize, peritoneal hypothermia increased door-to-balloon time, while achieving the target temperature did not reduce infarct size and was connected to poorer prognosis and, especially, to stent thrombosis, implying serious limitations of both the method and the study design [73,78].

Due to previous results showing benefit in anterior MI, Noc et al. designed a multi-center RCT, the COOL AMI EU pilot trial, that evaluated the safety and efficacy of therapeutic hypothermia with endovascular cooling in patients with anterior STEMI undergoing PCI [74]. Apart from the specific enrollment population, what was new in this trial was the introduction of the new ZOLL Proteus^TM^ Intravascular Temperature Management System^TM^ (ZOLL Medical Corporation, Chelmsford, MA, USA), which was more powerful and quick in achieving target temperature, regarding the time and energy required. The investigators enrolled 50 patients, randomizing them 1:1 either to endovascular cooling or control. All patients underwent successful primary stenting. This pilot study was powered for safety. IS was evaluated with CMR 4–6 days post-procedure and patients were followed-up for 30 days. It is important to remark that although a delay from randomization to balloon was reported in the hypothermia arm (59 vs. 42 min; *p* = 0.01), it was not the main reason of the overall increased ischemic time in the hypothermia group; this was mainly attributed to longer time from symptom onset to randomization (213 vs. 174 min; *p* = 0.08). Regarding safety, there were no statistically significant differences at 30 days follow-up, except for the report of self-terminating paroxysmal atrial fibrillation demonstrating numerical trend and not significance, in 32% of the patients in hypothermia arm vs. 8% in the control group (*p* = 0.074). Regarding efficacy, a statistically non-significant numerical 7.1% absolute and 30% relative reduction in IS was demonstrated. It was the first randomized trial that targeted only patients with anterior MI, with a newer and safer cooling device, achieving a 1.1 °C lower endovascular temperature, without any difference in procedure-related or 30-days adverse events, associated with a reduction in IS, urging the need of designing a pivotal trial, powered for efficacy.

COOL-MI InCor was another single-center RCT that aimed to evaluate the safety and efficacy of therapeutic hypothermia, via endovascular cooling, prior to PCI in patients with anterior and inferior MI [75]. Towards this purpose, they enrolled 50 patients (35 in the hypothermia arm and 15 in the control arm). Of them, 38% were presented with anterior MI and 64% with inferior MI. By using a more novel endovascular cooling system than in previous trials, the investigators achieved a reperfusion temperature of 33.1 °C ± 0.9 °C. Regarding safety, Dallan et al. demonstrated that mean door-to-ballon time was 92.1 ± 20.5 in the hypothermia arm and 87 ± 24.4 min in the control arm, with a non-significant difference of 5.1 min (*p* = 0.509), and the prevalence of MACEs, at 30 days follow-up period, did not differ between study arms (21.7% vs. 20%, *p* = 0.237). Moreover, mean IS, evaluated by CMR at 30 days post-procedure, was 13.9% ± 8% in the hypothermia arm vs. 13.8% ± 10.8% in the control arm (*p* = 0.801) and mean LVEF was 43.3% ± 11.2% vs. 48.3 ± 10.9%, respectively, *p* = 0.194, concluding that therapeutic hypothermia, although not more harmful, did not offer additional myocardial protection regarding IS or left ventricular function.

Following the confident results of the COOL AMI EU pilot trial in patients with anterior STEMI [74], Noc et al. designed and conducted the COOL AMI EU Pivotal Trial, a multi-center RCT, powered for efficacy, utilizing the ZOLL Proteus intravascular cooling system, in 111 patients (58 to hypothermia arm and 53 to control arm) with anterior STEMI undergoing PCI [76]. Endovascular cooling was successful in 95% of the hypothermia arm patients (55/58), with an achieved temperature of 33.0 ± 0.9 °C. The primary efficacy endpoint was IS, evaluated by CMR 4–6 days post-procedure, and the investigators demonstrated no significant difference between groups (IS as a percent of LV mass of 21.3% in the hypothermia arm and 20.0% in the control arm, *p* = 0.540). On the other hand, a significant delay in door-to-ballon time was observed between arms, (61 ± 21 vs. 32 ± 18 min, *p <* 0.001); regarding safety, although therapeutic hypothermia was not significantly associated with increased MACEs at 30 days (8.6% vs. 1.9%; *p* = 0.117), hypothermia was connected to increased frequency of cardiogenic shock (10.3% vs. 0%; *p* = 0.028) and arrhythmic events, especially paroxysmal atrial fibrillation (43.1% vs. 3.8%; *p <* 0.001). The COOL AMI EU Pivotal Trial was the first trial using the novel endovascular technique systems powered for efficacy but it failed to document any benefit in infarct size or arrhythmogenicity.

There have been reports of out-of-hospital initiation of endovascular hypothermia in patients with STEMI. Testori et al. designed and carried out STATIM, an RCT enrolling 101 patients with STEMI to a hypothermia and a control arm prior to them undergoing PCI [77]. However, hypothermia did not only increase door-to-ballon time by 14 min between arms (*p <* 0.01), but it failed to show a significant difference regarding the myocardial salvage index (0.37 ± 0.26 in the control arm vs. 0.43 ± 0.27 in the hypothermia group, *p* = 0.27).

## 4. Selective Intracoronary Hypothermia

The unfavorable results of systemic hypothermia for the reduction of IS and prevention of myocardial reperfusion injury were associated with a number of drawbacks, which are related to the systemic manner of achieving hypothermia. In specific, the systemic route necessitates increased pre-reperfusion time to allow sufficient myocardial cooling, thus increasing the ischemic time, which could be related to the neutral effects, while in multiple occasions, the target temperature was not reached, therefore compromising the cardioprotective effects of hypothermia. Considering the associated adverse effects of administering systematic hypothermia, as well as the lack of efficacy, it has been proposed that a more selective way of cooling would provide more benefit. Thus, the concept of selective intracoronary hypothermia was developed. The method of intracoronary hypothermia has been described by Otterspoor et al. [79]. In short, after crossing the lesion with a guidewire, an over-the-wire balloon is advanced and inflated at the site of the occlusion. Following this, a wire measuring the pressure and temperature is used and distally advanced. Hypothermia is achieved with continuous administration of saline, which is available both at room temperature and at 4 °C. The saline administered first is the room temperature one (occlusion phase, as the balloon is inflated), in order to achieve a distal temperature of approximately 6–8 °C below the body temperature. Deflation of the balloon follows (reperfusion phase) and the second infusion of saline at 4 °C continues, in order to maintain a distal coronary temperature of 4–6 °C below body temperature. Several preclinical, and more recently, clinical, studies have evaluated the safety and potential efficacy of intracoronary cooling in patients with ACS.

Kim et al. [80] was one of the first to investigate the safety and feasibility of achieving selective hypothermia in an animal model. They used open-chest pigs, with or without MI, on which the LAD was ligated, followed by administration of lactated Ringer’s solution at room temperature and at 15 °C, for 3 min. The temperature was recorded systemically, as well as in the ischemic and non-ischemic myocardial regions. No animal developed hemodynamic compromise or arrhythmias. The was a linear relationship between the intramyocardial temperature and infusion solution temperature/infusion rates, with the cooled solution and the faster infusion rate being associated with lower temperatures. Furthermore, distal intracoronary temperature correlated well with intramyocardial temperature. An average of 6–8 °C reduction in temperature was achieved, with no difference in the systemic or non-ischemic region temperature.

Moving further along, Otake et al. [81] evaluated selective intracoronary hypothermia in pigs, aiming to show any effect in myocardial necrosis. The study consisted of two substudies. In the first one, after 15 min of coronary artery occlusion with a balloon, pigs were either infused with either 4 °C or 36.5 °C normal saline at a rate of 2.5 mL/min for one hour. Reperfusion was started one hour after the occlusion, and thus, colling continued for 15 min after revascularization. The second investigation compared hypothermia (using the 4 °C saline at a rate of 8 mL/min for 30 min) with simple reperfusion, with cooling being started at the time of the reperfusion (60 min after the occlusion). In all instances, technical success was achieved and coronary flow reserve was preserved in all subjects. The investigators reported significantly decreased rates of ventricular arrhythmias and ratio of necrosis to ischemic area in the first substudy (9 ± 2 vs. 36 ± 4%; *p* < 0.0001), and a trend towards significance in the second substudy (33 ± 2 vs. 45 ± 5; *p* = 0.08). Interestingly, both cohorts had suppressed levels of 8-iso-prostaglndin F2α, which provides an explanation of the cardioprotective effects of hypothermia, i.e., attenuation of reperfusion-related oxidative stress.

The cardioprotection of intracoronary hypothermia has been, recently, also related to attenuation of mitochondrial injury. An ex vivo, isolated, beating pig heart study was conducted by El Farissi et al. [68] with a total of four hearts, two treated with intracoronary cooling followed by reperfusion and two with normal reperfusion strategies. Histology studies showed that, in comparison to hearts with normal reperfusion, hypothermia treatment resulted in a more frequently intact mitochondria presence, thus documenting the benefit of selective hypothermia in mitochondrial homeostasis.

The first clinical study to evaluate the safety and feasibility of selective intracoronary hypothermia in cooling was performed by Otterspoor et al. [79], with the method previously described in 10 patients undergoing pPCI (Table 2). Using this technique, target coronary temperature (6 °C below body temperature) was achieved in a median of 27 s (21–46), while all patients had normal left ventricular ejection fraction and successful pPCI. Two patients with inferior wall MI experienced transient conduction disturbances, while two other individuals had ventricular tachyarrhythmia episodes (one was reported prior to the procedure). No other side effects occurred, while systemic temperature remained unchanged.

Wang et al. [82] further assessed intracoronary hypothermia in 60 patients presenting with STEMI and having a thrombolysis in MI (TIMI) flow grade of 0/1. The primary endpoint was absolute IS, which was defined as the ratio of IS to myocardium at risk at 7 days post-pPCI using cardiac magnetic resonance. The technical success rate was 100%, with no arrhythmia or hemodynamic instability occurrence. The temperature was reduced by a mean of 5.8 ± 1.1 °C, while the time to achieve temperature was 31 ± 8 min and the door-to-flow delay (due to the cooling intervention) was 13 min. Absolute IS was significantly reduced in the hypothermia group (44.9 ± 5.9% vs. 50.7 ± 10.7%, *p* = 0.022) and particularly in those with anterior STEMI (46.1 ± 7.5% vs. 55.3 ± 11.2%, *p* = 0.023). No difference in adverse events post-revascularization were found at 30 days between groups. Biomarkers of myocardial injury after pPCI, including high-sensitivity troponin T and N-terminal prohormone of brain natriuretic peptide (NT-proBNP), were significantly reduced in the hypothermia group. Furthermore, a higher number of premature ventricular contractions and non-sustained ventricular tachycardia episodes were observed in the control group.

The largest, to date, analysis was recently published by El Farissi et al. [83]. The EURO-ICE trial enrolled 200 patients with anterior STEMI, which were randomized 1:1 to undergo either selective intracoronary hypothermia and pPCI or pPCI alone. The cooling included 7–10 min before reperfusion and 10 min after reperfusion. A total of 94 patients ultimately underwent the hypothermia procedure, and no difference was found in the technical success of the pPCI. The door-to-balloon time was 15 min longer in the intracoronary hypothermia arm [37 (IQR 33–44) versus 22 min (IQR 18–26); *p* < 0.001]. This is considerable, as the mean duration of the occlusion phase was 8 min, thus indicating an additional mean delay of 7 min for instrumentation, in order to perform the procedure. The time to target temperature was 43 (18–113) seconds. Regarding post-procedural outcomes, ventricular fibrillation occurred in nine hypothermia and seven control patients (*p* = 0.60), while atrial fibrillation occurred in zero versus three patients, respectively (*p* = 0.08), and hemodynamic instability occurred in three versus two patients, respectively (*p* = 0.65). However, in respect to the primary outcomes of IS as a percentage of left ventricular mass at 3 months, no difference was found between the two groups (23.1 ± 12.5% versus 21.6 ± 12.2%; *p* = 0.43). Additionally, no difference was found in regard to absolute IS and LVEF.

Despite showing more promise, compared to systemic hypothermia, as it overlooked known limitations of this approach and could limit systemic treatment-related adverse events whilst providing local cardioprotection, to date, the human data from selective intracoronary hypothermia seem disappointing, with only one study showing benefit and the key EURO-ICE trial failing to show any cardioprotective effect. Given the conflicting results, and considering the studies of systemic hypothermia, it could be possible that the methods and protocols of hypothermia currently used, despite being effective in animal studies, could be unsuccessful in humans and in the clinical conditions surrounding the clinical presentation of MI, compared to a controlled laboratory setting. Thus, there might be needed alterations in the timing, extent of cooling and duration, along with other methodological aspects, in order to identify any benefit in clinical studies. However, it should be recognized that, regarding the EURO-ICE trial, the authors note lower-than-historical-data IS and mortality, which could partially explain the neutral results. On the other hand, the larger door-to-balloon time in the cooling arm could also be related to these outcomes. It is positive, though, that the safety of the procedure is undoubted in these early studies, with low rates of procedure-related adverse events as well as arrhythmia occurrence. Further studies, considering the designs of previous trials and potentially with modifications of the current protocols after testing variations in large animal studies, are necessary in order to decide whether intracoronary hypothermia will be used in myocardial reperfusion injury prevention. However, for the time being, neither systemic nor intracoronary hypothermia can be recommended in patients with STEMI for this purpose.

## 5. Future Perspectives

**Figure 1:**
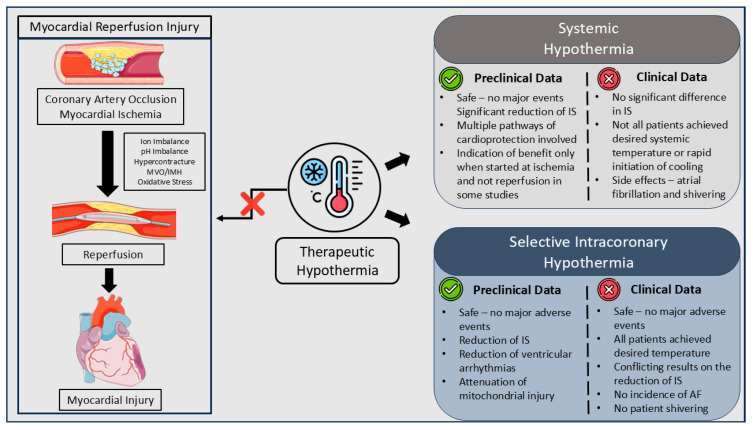
The effect of hypothermia in myocardial reperfusion injury. Abbreviations: IS: infarct size; MVO: microvascular obstruction; IMH: intramyocardial hemorrhage; AF: atrial fibrillation.

Systemic and intracoronary hypothermia add to a large amount of neutral evidence regarding myocardial reperfusion injury prevention and cardioprotection during STEMI with the use of additive to pPCI interventions. Despite the preclinical studies showing benefit of hypothermia in animal models of myocardial ischemia, through the years, this has failed to be translated into a clinical benefit (Figure 1). However, while noting the positive preclinical evidence, some investigators have postulated that cooling, in order to be effective, needs to be started during ischemia and not during reperfusion, in order to exert its cardioprotection [60]. This is more easily performed in preclinical, controlled setting, rather than in clinical studies, and, therefore, it may constitute a reason why there is no benefit, to date, shown in human data. Moreover, despite the progression of studies and understanding of previous pitfalls, such as the inclusion of only TIMI flow 0/1 patients and local intracoronary cooling in order to achieve the desired temperature, which were not regarded in early systemic hypothermia studies, other limitations, such as a uniform definition of myocardial injury prevention endpoints and IS reduction in all trials, accounting for collateral perfusion and ischemia time, the type of STEMI included, additive medications, comorbidities of patients (which are not present in animal studies), the duration of cooling period and the impact of door-to-balloon prolongation, still need to be addressed in following research efforts. Notably, only recently has a consensus on endpoints regarding cardioprotection trials been published, which should be used as guidance in future studies [84]. Currently, the STEMI-Cool study is ongoing (NCT06128993), and aims to evaluate the safety and efficacy of intracoronary hypothermia in blood biomarkers, invasive coronary hemodynamic tests and IS measured by CMR.

**Figure 2:**
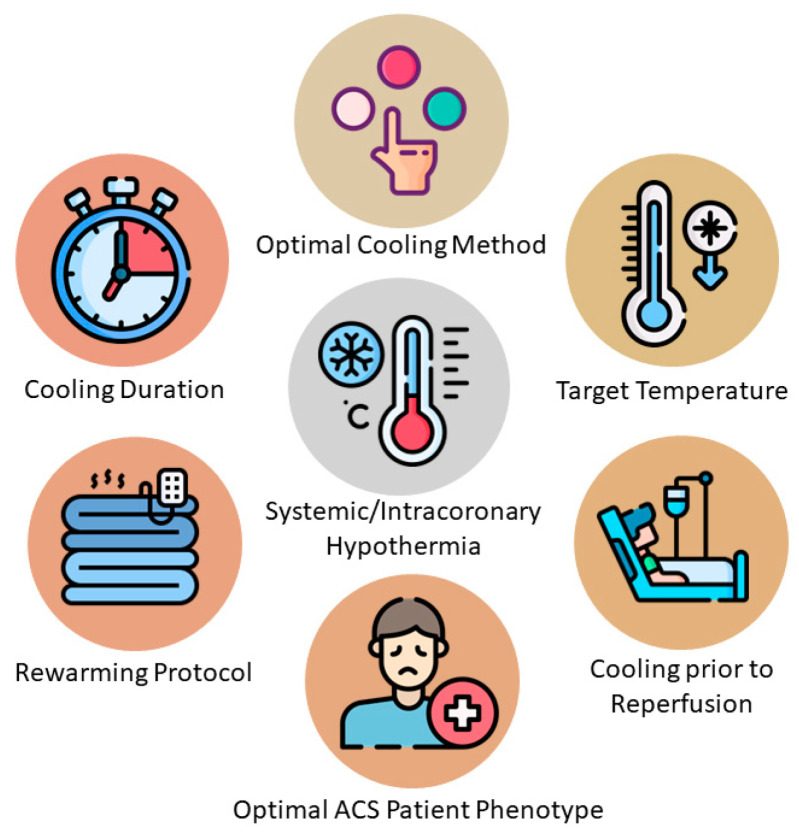
Limitations and unanswered questions of hypothermia studies. Abbreviations: ACS: acute coronary syndrome.

Awaiting further results, the studies available, to date, in both systemic and intracoronary hypothermia, do not support the routine use of such interventions in the catheterization laboratory along with pPCI, in order to limit IS and improve patient outcomes, as the majority of study results were neutral. This observation should be, however, contextualized based on the respective limitations of both interventions and performed studies, as already discussed. In specific, such limitations and unanswered questions include the non-standardized hypothermia and follow-up protocols, not achieving, in a timely manner, the target temperature (mostly with systemic methods) and the prolongation of ischemia time, as well as the need for the target temperature to be potentially achieved before reperfusion and the extent of hypothermia, its optimal duration and rewarming protocols (Figure 2). In order to inform clinical practice, further research is needed, while retrospection of these results, as well as advancements in protocols and technology, could probably allow to conduct enhanced protocols, which are less bound to pitfalls and limitations and could potentially provide more positive results.

Current intracoronary hypothermia methods involve the infusion of cooled saline with the use of the previously described methods. Along with designing trials considering the aforementioned limitations, data from animal studies have examined altered ways of intracoronary hypothermia, including infusion of autologous, cooled blood [85] and novel catheters [86]. In more detail, Pei et al. [86] assessed a novel device consisting of a cryogenic catheter, using nitrous oxide as a direct cooling media, in physiological and MI pig models for 30 min, initiating cooling at reperfusion. The average time to a lower temperature steady state was 4.8 ± 0.8 s, while they found a reduction in inflammatory molecules, as well as edema, IMH and MVO in the pigs that were treated with hypothermia. Furthermore, the ejection fraction was improved in the hypothermia pigs. Technological advancements and new options to deliver intracoronary hypothermia could be promising and further animal and human data are needed towards this direction.

Currently, the only approved intervention for cardioprotection in STEMI is the use of supersaturated oxygen (SSO2). SSO2 is delivered via a specific catheter, allowing to provide hyperbaric levels of oxygen in the coronary artery circulation, increasing PO2 of the blood in the coronary artery to approximately 900 mmHg, in order to increase oxygen delivery in myocardial and endothelial cells and, therefore, prevent myocardial necrosis and microvascular damage. This procedure is performed immediately after reperfusion, for 60 min. Early preclinical as well as randomized clinical studies have shown safety and efficacy in infarct size reduction and HF hospitalizations [87,88,89,90]. With the exception of this procedure, pharmacological (i.e., nitrates, adenosine, cyclosporine) and interventional (i.e., LV unloading, mechanical conditioning, coronary sinus occlusion) studies have failed to show similar to SSO2 benefit [91]. The advancement of the field requires further research, as well as the potential combination of cardioprotective interventions. Using combined SSO2 and intracoronary hypothermia, or intracoronary hypothermia with pharmacological agents, could be a new frontier of research in the evolving field of cardioprotection. Lastly, combining invasive hypothermia with pharmacological hypothermia [78,92], potentially with a locally delivered agent, could be an interesting hypothesis, given that more extended hypothermia is proven to be beneficial in patients.

## 6. Conclusions

Despite showing promise in preclinical studies, neither systemic nor selective intracoronary hypothermia shows extensive benefits in myocardial reperfusion injury following STEMI. Novel studies, considering the previous limitations, and research in hypothermia protocols and optimal patient phenotypes, as well as the combination of hypothermia with other invasive or pharmaceutical cardioprotective methods, could be of interest in the future, aiming to show a benefit of this intervention in the ACS population and reduce reperfusion-related complications.

## Figures and Tables

**Table 1:** Clinical studies of systemic hypothermia in acute coronary syndromes.

Trial	Design	n	Anterior MI	Hypothermia Technique	Target Temperature (°C)	Achieved Temperature (°C)	Door-to-Ballon Time (min) (Hypothermia vs. Control)	Patients Achieved Target Temperature	Imaging	IS (% of LVM) (Hypothermia vs. Control)	Safety (Hypothermia vs. Control)
COOL-MI I pilot [69]	Multi-center, prospective RCT	42	45%	Endovascular cooling	33	33.2 ± 0.9	87 ± 30 vs. 104 ± 44	20/21	SPECT	2% vs. 8% (*p* = 0.8)	MACEs (30 days) 0% vs. 10% (*p* = NS)
NICAMI [70]	Single-arm, open-label, feasibility	9	33%	Noninvasive surface cooling	34.5	N/R	38	9/9	SPECT	23%	No hemodynamic or arrhythmic instability
RAPID-MI-ICE [71]	Single-center, prospective RCT	20	72%	Endovascular cooling + cold saline infusion	35	34.7 ± 0.3	43 ± 7 vs. 40 ± 6	9/10	CMR	13.7% vs. 20.5% (*p* = 0.08)	MACEs (30 days) 0% vs. 0% (*p* = NS)
CHILL-MI [72]	Multi-center, prospective RCT	120	42%	Endovascular cooling + cold saline infusion	33	34.7	42 ± 16 vs. 33 ± 21	46/60	CMR	IS/MaR: 40.5% vs. 46.6% (*p* = 0.15)	No significant difference in thrombotic or bleeding events
VELOCITY [73]	Multi-center, prospective RCT	54	46%	Peritoneal lavage	32.5	34.0 ± 0.8	62 ± 15 vs. 47 ± 9 (*p* = 0.007)	24/28	CMR	17.2% ± 2.3 vs. 16.0% ± 6.1 (*p* = 0.54) IS/MaR: 67.3% vs. 55.8% (*p* = 0.36)	MACEs (30 days) 6 (21.4%) vs. 0 (0%) patients (*p* = 0.01)
COOL AMI EU pilot [74]	Multi-center, prospective RCT	50	100%	Endovascular cooling + cold saline infusion	32	33.6 ± 1.0	59 ± 19 vs. 42 ± 23	23/25	CMR	16.7% vs. 23.8% (*p* = 0.31)	No difference in adverse events PAF (32% versus 8%; *p* = 0.074)
COOL-MI InCor [75]	Single-center, prospective RCT	50	38%	Endovascular cooling + cold saline infusion	32	33.1 ± 1.0	92.1 ± 20.5 vs. 87 ± 24.4	35/35	CMR	13.9% vs. 13.8% (*p* = 0.801)	No difference: -All-cause mortality (2.9% vs. 6.7%, *p* = 0.237) -MACEs (21.7% vs. 20%, *p* = 0.237)
COOL AMI EU Pivotal [76]	Multi-center, prospective RCT	111	100%	Endovascular cooling + cold saline infusion	32	33.0 ± 0.9	61 ± 21 vs. 32 ± 18	55/58	CMR	21.3% vs. 20.0% (*p* = 0.540)	Non-significant increase of MACEs (8.6% vs. 1.9%; *p* = 0.117) Significant difference in: -cardiogenic shock (10.3% vs. 0%, *p* = 0.028) -PAF (43.1% vs. 3.8%, *p* < 0.001)
STATIM [77]	Single-center, prospective RCT	101	51%	Endovascular cooling + cold saline infusion	34	34.4 ± 0.6	103 ± 21 vs. 89 ± 24	38/47	CMR	MSI: 0.43 ± 0.27 vs. 0.37 ± 0.26 (*p* = 0.27).	No difference in MACEs Numerical trend towards increased bleeding events in the hypothermia arm

CMR: cardiac magnetic resonance, IS: infarct size, LVM: left ventricle mass, MaR: myocardium at risk, MACEs: major adverse cardiovascular events, MI: myocardial infarction, MSI: myocardial salvage index, N/R: not reported, NS: non-significant, PAF: paroxysmal atrial fibrillation, RCT: randomized controlled trial, SPECT: single-photon emission computed tomography.

**Table 2:** Studies on intracoronary hypothermia in acute coronary syndromes.

Study	Design	Population	Number of Participants	TIMI 0/1	Infarct Size Imaging Method	Door to Balloon Time (minutes)	Ischemic Time Prolongation (minutes)	Time to Achieve-Target Temperature (seconds)	Target Temperature Achieved (%)	Mortality	Infarct Size	Arrhythmia Outcomes	Stent Thrombosis
Otterspoor et al. [79]	Observational, Feasibility	STEMI (60% Anterior, 40% Inferior)	10	8	N/R	55 (46–57)	19.2 (18.4–19.6)	27 (21–46)	100%	0%	N/R	20% AV block 20% ventricular tachyarrhythmia	10%
Wang et al. [82]	Observational	STEMI (54% Anterior, 46% Inferior)	60 (30 intervention, 30 control)	60	CMR, after 7 days	96 ± 24 vs. 83 ± 32 (*p* = 0.07)	13	31 ± 8	100%	7% vs. 10% (30 days)	Mean IS/MaR: 44.85 ± 5.89% vs. 50.69 ± 10.75%; =0.022 Mean IS/LVM: 18.76 ± 7.61 vs. 23.64 ± 10.08; *p* = 0.059	Non-sustained VT: 0%	3% vs. 3%
El Farissi et al. [83]	Randomized Controlled Trial	Anterior STEMI	200 (100 intervention, 100 control)	200	CMR, after 3 months	37 (33–44) vs. 22 (18–26); *p* < 0.001	15	43 (18–113)	100%	1% vs. 0% (3 months)	IS (%LVM): 23.1 ± 12.5 vs. 21.6 ± 12.2; *p* = 0.43 IS (g): 26.1 ± 17.8 vs. 24.5 ± 15.7; *p* = 0.52 Myocardial salvage index: 0.54 ± 0.24 vs. 0.55 ± 0.25; *p* = 0.82	VT: 9 vs. 7 patients (*p* = 0.60) AF: 0 vs. 3 patients (*p* = 0.08)	2 vs. 1 patients (*p* = 0.56)

Abbreviations: STEMI: ST-elevation myocardial infarction; TIMI: thrombolysis in myocardial infarction; AV: atrioventricular; CMR: cardiac magnetic resonance; IS: infarct size; MaR: myocardium at risk; LVM; left ventricular mass; VT: ventricular tachycardia; AF: atrial fibrillation; g: grams; N/R: not reported.

## Data Availability

No new data were created, as this is a review article.
